# Supplementary material for: Unveiling the Bioactive Potential of the Invasive Jellyfish Phyllorhiza punctata Through Integrative Transcriptomic and Proteomic Analyses
Source: Biomolecules. 2025 Aug 4;15(8):1121. doi: 10.3390/biom15081121 (PMC12383608; doi:10.3390/biom15081121)
Supplement: Supplementary file 1 [file biomolecules-15-01121-s001.zip › Suplementary Material/FileS3/PEPFOLD4/XP_065072136_4667/clusters.html]

 PEP-FOLD clusters


Download archive of all models  here   

|  |  |  |
| --- | --- | --- |
| # | file | sOPEP |
| # cluster 1 - Size: 11 |  |  |
|  | XP\_065072136\_4667-model1 | -19.7349 |
|  | XP\_065072136\_4667-model1.1 | -19.3489 |
|  | XP\_065072136\_4667-model1.2 | -19.3088 |
|  | XP\_065072136\_4667-model1.3 | -19.3088 |
|  | XP\_065072136\_4667-model1.4 | -19.2747 |
|  | XP\_065072136\_4667-model1.5 | -19.1632 |
|  | XP\_065072136\_4667-model1.6 | -19.1632 |
|  | XP\_065072136\_4667-model1.7 | -19.1444 |
|  | XP\_065072136\_4667-model1.8 | -19.1444 |
|  | XP\_065072136\_4667-model1.9 | -19.1371 |
|  | XP\_065072136\_4667-model1.10 | -19.0489 |
| # cluster 2 - Size: 9 |  |  |
|  | XP\_065072136\_4667-model2 | -19.5047 |
|  | XP\_065072136\_4667-model2.1 | -19.3348 |
|  | XP\_065072136\_4667-model2.2 | -19.3348 |
|  | XP\_065072136\_4667-model2.3 | -19.2438 |
|  | XP\_065072136\_4667-model2.4 | -19.2304 |
|  | XP\_065072136\_4667-model2.5 | -19.2301 |
|  | XP\_065072136\_4667-model2.6 | -19.2301 |
|  | XP\_065072136\_4667-model2.7 | -19.2291 |
|  | XP\_065072136\_4667-model2.8 | -19.101 |
| # cluster 3 - Size: 3 |  |  |
|  | XP\_065072136\_4667-model3 | -19.4833 |
|  | XP\_065072136\_4667-model3.1 | -19.2144 |
|  | XP\_065072136\_4667-model3.2 | -19.1449 |
| # cluster 4 - Size: 8 |  |  |
|  | XP\_065072136\_4667-model4 | -19.4829 |
|  | XP\_065072136\_4667-model4.1 | -19.4106 |
|  | XP\_065072136\_4667-model4.2 | -19.3297 |
|  | XP\_065072136\_4667-model4.3 | -19.2897 |
|  | XP\_065072136\_4667-model4.4 | -19.2603 |
|  | XP\_065072136\_4667-model4.5 | -19.1038 |
|  | XP\_065072136\_4667-model4.6 | -19.0636 |
|  | XP\_065072136\_4667-model4.7 | -18.981 |
| # cluster 5 - Size: 2 |  |  |
|  | XP\_065072136\_4667-model5 | -19.4777 |
|  | XP\_065072136\_4667-model5.1 | -19.3642 |
| # cluster 6 - Size: 5 |  |  |
|  | XP\_065072136\_4667-model6 | -19.404 |
|  | XP\_065072136\_4667-model6.1 | -19.2808 |
|  | XP\_065072136\_4667-model6.2 | -19.2408 |
|  | XP\_065072136\_4667-model6.3 | -19.2205 |
|  | XP\_065072136\_4667-model6.4 | -18.9904 |
| # cluster 7 - Size: 9 |  |  |
|  | XP\_065072136\_4667-model7 | -19.365 |
|  | XP\_065072136\_4667-model7.1 | -19.2457 |
|  | XP\_065072136\_4667-model7.2 | -19.2286 |
|  | XP\_065072136\_4667-model7.3 | -19.2132 |
|  | XP\_065072136\_4667-model7.4 | -19.1899 |
|  | XP\_065072136\_4667-model7.5 | -19.1899 |
|  | XP\_065072136\_4667-model7.6 | -19.1705 |
|  | XP\_065072136\_4667-model7.7 | -19.0974 |
|  | XP\_065072136\_4667-model7.8 | -19.0178 |
| # cluster 8 - Size: 20 |  |  |
|  | XP\_065072136\_4667-model8 | -19.3583 |
|  | XP\_065072136\_4667-model8.1 | -19.3583 |
|  | XP\_065072136\_4667-model8.2 | -19.3131 |
|  | XP\_065072136\_4667-model8.3 | -19.2698 |
|  | XP\_065072136\_4667-model8.4 | -19.2641 |
|  | XP\_065072136\_4667-model8.5 | -19.2641 |
|  | XP\_065072136\_4667-model8.6 | -19.2511 |
|  | XP\_065072136\_4667-model8.7 | -19.2176 |
|  | XP\_065072136\_4667-model8.8 | -19.1922 |
|  | XP\_065072136\_4667-model8.9 | -19.181 |
|  | XP\_065072136\_4667-model8.10 | -19.181 |
|  | XP\_065072136\_4667-model8.11 | -19.1763 |
|  | XP\_065072136\_4667-model8.12 | -19.1653 |
|  | XP\_065072136\_4667-model8.13 | -19.1308 |
|  | XP\_065072136\_4667-model8.14 | -19.114 |
|  | XP\_065072136\_4667-model8.15 | -19.052 |
|  | XP\_065072136\_4667-model8.16 | -19.0378 |
|  | XP\_065072136\_4667-model8.17 | -18.9883 |
|  | XP\_065072136\_4667-model8.18 | -18.9883 |
|  | XP\_065072136\_4667-model8.19 | -18.9859 |
| # cluster 9 - Size: 8 |  |  |
|  | XP\_065072136\_4667-model9 | -19.356 |
|  | XP\_065072136\_4667-model9.1 | -19.3093 |
|  | XP\_065072136\_4667-model9.2 | -19.1665 |
|  | XP\_065072136\_4667-model9.3 | -19.1517 |
|  | XP\_065072136\_4667-model9.4 | -19.1148 |
|  | XP\_065072136\_4667-model9.5 | -19.0764 |
|  | XP\_065072136\_4667-model9.6 | -19.0485 |
|  | XP\_065072136\_4667-model9.7 | -19.044 |
| # cluster 10 - Size: 11 |  |  |
|  | XP\_065072136\_4667-model10 | -19.3492 |
|  | XP\_065072136\_4667-model10.1 | -19.3137 |
|  | XP\_065072136\_4667-model10.2 | -19.3137 |
|  | XP\_065072136\_4667-model10.3 | -19.2913 |
|  | XP\_065072136\_4667-model10.4 | -19.2842 |
|  | XP\_065072136\_4667-model10.5 | -19.1813 |
|  | XP\_065072136\_4667-model10.6 | -19.1227 |
|  | XP\_065072136\_4667-model10.7 | -19.0953 |
|  | XP\_065072136\_4667-model10.8 | -19.07 |
|  | XP\_065072136\_4667-model10.9 | -19.0519 |
|  | XP\_065072136\_4667-model10.10 | -19.0064 |
| # cluster 11 - Size: 2 |  |  |
|  | XP\_065072136\_4667-model11 | -19.333 |
|  | XP\_065072136\_4667-model11.1 | -19.1343 |
| # cluster 12 - Size: 6 |  |  |
|  | XP\_065072136\_4667-model12 | -19.3199 |
|  | XP\_065072136\_4667-model12.1 | -19.3132 |
|  | XP\_065072136\_4667-model12.2 | -19.3132 |
|  | XP\_065072136\_4667-model12.3 | -19.1606 |
|  | XP\_065072136\_4667-model12.4 | -19.1049 |
|  | XP\_065072136\_4667-model12.5 | -19.0769 |
| # cluster 13 - Size: 6 |  |  |
|  | XP\_065072136\_4667-model13 | -19.2066 |
|  | XP\_065072136\_4667-model13.1 | -19.2014 |
|  | XP\_065072136\_4667-model13.2 | -19.1763 |
|  | XP\_065072136\_4667-model13.3 | -19.1763 |
|  | XP\_065072136\_4667-model13.4 | -19.1427 |
|  | XP\_065072136\_4667-model13.5 | -19.0083 |
|  |  |  |
